# A Mendelian Randomization Approach Using 3-HMG-Coenzyme-A Reductase Gene Variation to Evaluate the Association of Statin-Induced Low-Density Lipoprotein Cholesterol Lowering With Noncardiovascular Disease Phenotypes

**DOI:** 10.1001/jamanetworkopen.2021.12820

**Published:** 2021-06-07

**Authors:** Ge Liu, Mingjian Shi, Jonathan D. Mosley, Chunhua Weng, Yanfei Zhang, Ming Ta Michael Lee, Gail P. Jarvik, Hakon Hakonarson, Bahram Namjou-Khales, Patrick Sleiman, Yuan Luo, Frank Mentch, Joshua C. Denny, MacRae F. Linton, Wei-Qi Wei, C. Michael Stein, QiPing Feng

**Affiliations:** 1 Department of Biomedical Informatics, Vanderbilt University Medical Center, Nashville, Tennessee; 2 Division of Clinical Pharmacology, Department of Medicine, Vanderbilt University Medical Center, Nashville, Tennessee; 3 Department of Biomedical Informatics, Columbia University Medical Center, New York, New York; 4 Genomic Medicine Institute, Geisinger Health System, Danville, Pennsylvania; 5 Musculoskeletal Institute, Geisinger, Danville, Pennsylvania; 6 Department of Medicine, Division of Medical Genetics, University of Washington Medical Center, Seattle; 7 Department of Genome Sciences, University of Washington, Seattle; 8 The Center for Applied Genomics, Children’s Hospital of Philadelphia, Philadelphia, Pennsylvania; 9 Department of Pediatrics, The Perelman School of Medicine, University of Pennsylvania, Philadelphia; 10 Division of Human Genetics, Children's Hospital of Philadelphia, Philadelphia, Pennsylvania; 11 Division of Pulmonary Medicine, Children's Hospital of Philadelphia, Philadelphia, Pennsylvania; 12 UC Department of Pediatrics, Cincinnati Children's Hospital Medical Center, Cincinnati, Ohio; 13 Department of Preventive Medicine, Feinberg School of Medicine, Northwestern University, Chicago, Illinois; 14 at the time of the study, Department of Medicine, Vanderbilt University Medical Center, Nashville, Tennessee; 15 All of Us Research Program, National Institutes of Health, Bethesda, Maryland; 16 now, National Institutes of Health, Bethesda, Maryland; 17 Division of Cardiovascular Medicine, Department of Medicine, Vanderbilt University Medical Center, Nashville, Tennessee; 18 Department of Pharmacology, Vanderbilt University, Nashville, Tennessee

## Abstract

**Importance:**

Observational studies suggest that statins, which inhibit 3-hydroxy-3-methylglutaryl coenzyme A (HMG-CoA) reductase, may be associated with beneficial effects in many noncardiovascular diseases.

**Objective:**

To construct a weighted HMG-CoA reductase (*HMGCR*) gene genetic risk score (GRS) using variants in the *HMGCR* gene affecting low-density lipoprotein cholesterol as an instrumental variable for mendelian randomization analyses to test associations with candidate noncardiovascular phenotypes previously associated with statin use in observational studies.

**Design, Setting, and Participants:**

This cohort study included 53 385 unrelated adults of European ancestry with genome-wide genotypes available from BioVU (a practice-based biobank, used for discovery) and 30 444 unrelated adults with European ancestry available in the Electronic Medical Records and Genomics (eMERGE; a research consortium that conducts genetic research using electronic medical records, used for replication). The study was conducted from February 6, 2015, through April 31, 2019; data analysis was performed from August 26, 2019, through December 22, 2020.

**Interventions:**

An *HMGCR* GRS was calculated.

**Main Outcomes and Measures:**

The association between the *HMGCR* GRS and the presence or absence of 22 noncardiovascular phenotypes previously associated with statin use in clinical studies.

**Results:**

Of the 53 385 individuals in BioVU, 29 958 (56.1%) were women; mean (SD) age was 59.9 (15.6) years. The finding between the *HMGCR* GRS and the noncardiovascular phenotypes of interest in this cohort was significant only for type 2 diabetes. An *HMGCR* GRS equivalent to a 10-mg/dL decrease in the low-density lipoprotein cholesterol level was associated with an increased risk of type 2 diabetes (odds ratio [OR], 1.09; 95% CI, 1.04-1.15; *P* = 5.58 × 10^−4^). The *HMGCR* GRS was not associated with other phenotypes; the closest were increased risk of Parkinson disease (OR, 1.30; 95% CI, 1.07-1.58; *P* = .007) and kidney failure (OR, 1.18; 95% CI, 1.05-1.34; *P* = .008). Of the 30 444 individuals in eMERGE, 16 736 (55.0%) were women; mean (SD) age was 68.7 (15.4) years. The association between the *HMGCR* GRS and type 2 diabetes was replicated in this cohort (OR, 1.09; 95% CI, 1.01-1.17; *P* = .02); however, the *HMGCR* GRS was not associated with Parkinson disease (OR, 0.93; 95% CI, 0.75-1.16; *P* = .53) and kidney failure (OR, 1.18; 95% CI, 0.98-1.41; *P* = .08) in the eMERGE cohort.

**Conclusions and Relevance:**

A mendelian randomization approach using variants in the *HMGCR* gene replicated the association between statin use and increased type 2 diabetes risk but provided no strong evidence for pleiotropic effects of statin-induced decrease of the low-density lipoprotein cholesterol level on other diseases.

## Key Points

**Question:**

Are there associations between candidate noncardiovascular diseases and statin treatment using genetic variants in the 3-hydroxy-3-methylglutaryl coenzyme A reductase (*HMGCR*) gene that decrease low-density lipoprotein cholesterol levels as an instrumental variable?

**Findings:**

In a mendelian randomization approach cohort study including 53 385 individuals, using variants in the *HMGCR* gene affecting low-density lipoprotein cholesterol levels replicated the association between statin use and increased type 2 diabetes risk. However, no strong associations between 3-hydroxy-3-methylglutaryl coenzyme A reductase inhibition by statins and other diseases were noted.

**Meaning:**

In this cohort study, statin treatment was associated with an increased risk of type 2 diabetes, but there appeared to be no strong indication of other pleiotropic outcomes of the low-density lipoprotein cholesterol level–decreasing outcomes with this drug class on the clinical phenotypes studied.

## Teaser

This cohort study uses a mendelian randomization approach with variants in the *HMGCR* gene to evaluate associations between statin therapy and noncardiovascular diseases.

## Introduction

Statins (HMG coenzyme A [HMG-CoA] reductase inhibitors) are first-line medications for lowering circulating low-density lipoprotein cholesterol (LDL-C) levels, and they reduce the risk of cardiovascular disease (CVD) substantially.^1^ However, statins have also been reported to have many beneficial and deleterious associations with the risk of non-CVD conditions, suggesting that they have multiple effects of therapeutic importance. More than a quarter of US adults aged 40 years or older use statin therapy to prevent CVD.^2^ Given this widespread use, it is important to understand the spectrum of both deleterious and beneficial effects of long-term statin treatment in diseases and conditions beyond the cardiovascular system.

In addition to their effects on CVD, statins may have associations with other diseases (ie, pleiotropy) with both increases (eg, type 2 diabetes, myopathy, and rhabdomyolysis) and decreases (eg, cancers and infection) reported in the risk of many diseases.^3^ However, most studies reporting statin associations with diseases other than CVD are inconclusive. A comprehensive review of meta-analyses of 112 observational studies and 144 randomized clinical trials evaluated the strength of evidence for 278 noncardiovascular outcomes reported to be associated with statins.^3^ The authors found none of these statin-disease associations to be conclusive; however, 23 associations were suggestive or highly suggestive, requiring additional study.

Studies to evaluate statins and the risk of non-CVD are challenging. Placebo-controlled, randomized clinical trials of statins in CVD are generally of short duration and thus often unable to address the effects of long-term statin therapy on other diseases. Observational studies, on the other hand, are faced with a methodologic challenge—patients who receive statins are substantially different from those who do not—thus, any differences in outcomes observed between patients using and not using statin therapy could be due to residual confounding. Mendelian randomization (MR) is an approach that might provide less-biased insights.

An MR approach to study statin therapy makes use of the fact that there are naturally occurring variants in the gene *HMGCR* (OMIM 142910) that encodes the enzyme, HMG-CoA reductase, that statins target. Because those genetic variants are inherited randomly and remain unchanged, an MR approach may be less-vulnerable to reverse causation and residual confounding and might provide evidence consistent with a causal effect between statin treatment and candidate outcomes. This approach has shown an association between statin treatment and an increased risk of type 2 diabetes.^4^

The availability of deidentified genetic and clinical information in large biobanks provides the opportunity to use an MR approach to define the outcomes of modulating HMG-CoA activity associated with the risk of many diseases. Herein, we apply an MR approach using genetic variants in *HMGCR* in 2 large, practice-based biobanks: the biobank at Vanderbilt University (BioVU) and the Electronic Medical Records and Genomics (eMERGE) network. We constructed a weighted genetic risk score (GRS) for *HMGCR* using variants that affect LDL-C levels and tested the association of the GRS with 22 candidate outcomes previously reported to be associated with statins.

## Methods

The study was conducted from February 6, 2015, through April 31, 2019; data analysis was performed from August 26, 2019, through December 22, 2020. The study was approved by Vanderbilt University Medical Center institutional review board, which waived the need for informed consent owing to the use of deidentified data. This study followed the Strengthening the Reporting of Observational Studies in Epidemiology (STROBE) reporting guideline.

Data were obtained from BioVU, a deidentified DNA biobank, linked to the deidentified electronic health record (EHR) for patients at Vanderbilt University Medical Center. BioVU has been described in detail previously.^5,6,7^ Information available in BioVU includes diagnostic and procedure codes, demographic characteristics, clinical notes, problem lists, laboratory test values, and medications.^8,9,10^ We included adults aged 18 years and older of European ancestry with genome-wide genotypes available.

The eMERGE network is a consortium that uses DNA biorepositories with EHR systems for large-scale, high-throughput genetic research and has been described previously.^11,12,13^ We replicated BioVU findings in eMERGE data on adults of European ancestry aged 18 years and older after removing individuals common to both cohorts.

### Genetic Analysis

We selected candidate disease phenotypes that have previously been associated with statins in randomized clinical trials on the drugs or observational studies.^3^ In both BioVU and eMERGE cohorts, we established the presence or absence of 22 noncardiovascular phenotypes. We selected only phenotypes with more than 100 cases in BioVU to maintain statistical power and those that could be defined using PheCodes. For example, disease-associated mortality was not studied because defining such phenotypes in the EHR would require development and validation of a case-by-case protocol. We identified phenotypes using PheCodes, a phenotyping system based on clinical diagnosis codes (*International Classification of Diseases, Ninth Revision, Clinical Modification* [*ICD-9-CM*] and *International Statistical Classification of Diseases, 10th Revision, Clinical Modification* [*ICD-10-CM*]).^14,15^ A PheCode amalgamates related *ICD* codes mapping to a distinct disease or trait.^14,15^ A case was defined as an individual with 2 or more occurrences of the PheCode of interest in the EHR. Controls were individuals without that code. Individuals with 1 mention of the code or with related codes were excluded from the analysis to limit misclassification.

To evaluate possible associations between statin treatment and noncardiovascular effects (ie, pleiotropy), we used a previously reported genetic instrument for *HMGCR*.^16^ Specifically, we constructed a weighted GRS of *HMGCR* using 6 genetic variants within the *HMGCR* region that have been independently associated with LDL-C levels (eTable 1 in the Supplement). This *HMGCR* GRS is an instrumental variable not for statin use (ie, likelihood of receiving a statin) but rather for the probability of a statin effect (ie, lower LDL-C level). In other words, individuals with an *HMGCR* GRS that estimated the probability of lower enzyme activity would estimate the probability of the effects of statin therapy in a population. We also constructed an *HMGCR* GRS in the eMERGE cohort, using 5 of 6 single-nucleotide variants (rs2006760 was not successfully imputed in the eMERGE cohort and was therefore excluded from analysis). We calculated the *HMGCR* GRS by summing LDL-C increasing alleles, weighted by the effect size, but to reflect the same directional effects as statin therapy, we adjusted the *HMGCR* GRS to reflect a standard decrement of 10 mg/dL (to convert to millimoles per liter, multiply by 0.0259) so that a high GRS would be associated with lower LDL-C levels, as occurs with statin therapy.

### Statistical Analysis

We conducted logistic regression in assessing the association between the *HMGCR* GRS and candidate phenotypes using R, version 3.6.0 (R Foundation for Statistical Computing). We included age at the most recent medical encounter, sex, and length of EHR (defined as the time between each patient’s first and most recent medical encounter) as covariates for all candidate phenotype analyses, except for cancer of the prostate (PheCode 185), analyzed only in men, and miscarriage (PheCode 634), analyzed only in women. In addition, 10 principal components were used for adjustment in BioVU and 5 principal components for eMERGE, as available. For each candidate phenotype, we estimated odds ratios (ORs) and 95% CIs per decrease of 10 mg/dL in LDL-C levels between the *HMGCR* GRS and the phenotype of interest. For the 22 prespecified phenotypes, *P* < .002 (05/22 phenotypes) was considered significant in the BioVU cohort. In the eMERGE replication cohort, a Bonferroni-adjusted value of *P* < .05 was considered significant. All statistical tests were 2-sided. We also conducted Pearson correlation coefficient testing using R, version 3.6.0 (R Foundation for Statistical Computing).

In sensitivity analyses, we adjusted for statin use, with individuals receiving statin therapy defined as patients with 1 or more exposure to a statin documented in the EHR. In addition, we stratified the *HMGCR* GRS into quartiles and compared individuals in lower LDL-C level quartiles (estimated by *HMGCR* GRS) with those in the highest estimated LDL-C level quartile. Genotype quality control and imputation are described in the eMethods in the Supplement.

## Results

### The BioVU Discovery Cohort

We calculated an *HMGCR* GRS for 53 385 unrelated adults of European ancestry in BioVU who had a mean (SD) age of 59.93 (15.61) years; a total of 29 958 women (56.11%) and 23 427 men (43.89%) were included. The mean (SD) length of available EHRs was 10.24 (7.01) years (Table 1).

**Table 1.** Cohort Demographic Characteristics

Characteristic	Patients, mean (SD)	
	BioVU (n = 53 385)	eMERGE (n = 30 444)
Sex, No. (%)		
Women	29 958 (56.11)	16 736 (55.0)
Men	23 427 (43.89)	13 708 (45.0)
Age, y	59.93 (15.61)	68.65 (15.43)
EHR length, y	10.24 (7.01)	17.11 (9.89)
Statin therapy, No. (%) of patients	25 625 (48.0)	16 060 (52.8)

Abbreviations: BioVU, biobank at Vanderbilt University; EHR, electronic health record; eMERGE, Electronic Medical Records and Genomics network.

As expected, the *HMGCR* GRS was associated with a diagnosis of hypercholesterolemia. For a 10-mg/dL *HMGCR* GRS–estimated decrease of the LDL-C level, the OR of having the PheCode of hypercholesterolemia was 0.91 (95% CI, 0.85-0.98; *P* = .009) (eTable 2 and the eFigure in the Supplement). In a subset of patients (n = 29 652) with both *HMGCR* GRS and measured LDL-C levels, the *HMGCR* GRS correlated with measured LDL-C levels (correlation coefficient, −0.03; *P* = 5.60 × 10^−8^).

The finding between the *HMGCR* GRS and the prespecified noncardiovascular phenotypes of interest was significant only for type 2 diabetes (Table 2) (OR, 1.10; 95% CI, 1.04-1.15; *P* = 5.58 × 10^−4^). The finding was also significant after further adjusting for statin use (OR, 1.13; 95% CI, 1.07-1.19; *P* = 8.50 × 10^−6^) (eTable 3 in the Supplement).

**Table 2.** Findings for *HMGCR* Genetic Risk Score and Candidate Noncardiovascular Phenotypes in the Biobank at Vanderbilt University^a^

PheCode	Description	Odds ratio (95% CI)	*P* value	No. (%)		
				Total	Cases	Controls
250.2	Type 2 diabetes	1.10 (1.04-1.15)	5.58 × 10^−4^	47 757	9210 (19.3)	38 547 (80.7)
332	Parkinson disease	1.30 (1.07-1.58)	.007	41 193	553 (1.3)	40 640 (98.7)
585.32	Kidney disease	1.18 (1.05-1.34)	.008	39 811	1308 (3.3)	38 503 (96.7)
772.4	Rhabdomyolysis	1.38 (0.96-1.97)	.08	46 140	159 (0.3)	45 981 (99.7)
202.2	Non-Hodgkin lymphoma	1.21 (0.96-1.52)	.11	51 062	367 (0.7)	50 695 (99.3)
151	Cancer of stomach	0.86 (0.71-1.05)	.13	45 818	509 (1.1)	45 309 (98.9)
800.1	Fracture of neck or femur	1.11 (0.93-1.31)	.24	45 613	679 (1.5)	44 934 (98.5)
770	Myalgia and myositis, unspecified	1.05 (0.96-1.13)	.28	50 460	3202 (6.3)	47 258 (93.7)
185	Cancer of prostate	0.94 (0.84-1.06)	.31	18 262	1786 (9.8)	16 476 (90.2)
577.1	Acute pancreatitis	1.09 (0.91-1.31)	.33	51 731	596 (1.2)	51 135 (98.8)
38	Septicemia	1.04 (0.96-1.13)	.38	46 724	3049 (6.5)	43 675 (93.5)
634	Miscarriage; stillbirth	0.88 (0.65-1.18)	.39	27 815	234 (0.8)	27 581 (99.2)
8.52	Intestinal infection due to *Clostridioides difficile*	0.94 (0.79-1.10)	.43	51 807	672 (1.3)	51 135 (98.7)
585.1	Acute kidney failure	1.02 (0.96-1.09)	.46	44 949	6446 (14.3)	38 503 (85.7)
70.3	Viral hepatitis C	0.96 (0.85-1.10)	.58	44 776	1168 (2.6)	43 608 (97.4)
366.2	Senile cataract	1.02 (0.95-1.09)	.67	50 734	4469 (8.8)	46 265 (91.2)
290.1	Dementias	1.03 (0.89-1.20)	.68	42 515	890 (2.1)	41 625 (97.9)
153.2	Colon cancer	0.98 (0.88-1.10)	.77	43 091	1510 (3.5)	41 581 (96.5)
38.3	Bacteremia	0.99 (0.89-1.10)	.83	45 543	1868 (4.1)	43 675 (95.9)
155.1	Malignant neoplasm of liver, primary	1.02 (0.83-1.25)	.84	45 770	461 (1.0)	45 309 (99.0)
359.2	Myopathy	0.99 (0.86-1.13)	.86	47 572	1028 (2.2)	46 544 (97.8)
743.11	Osteoporosis	1.00 (0.92-1.08)	.97	45 863	3410 (7.4)	42 453 (92.6)

^a^ The analyses were adjusted for sex, age at most recent visit, electronic health record length, and 10 principal components for ancestry. Cancer of the prostate was analyzed only in men; miscarriage or stillbirth was analyzed only in women. The *HMGCR* GRS was standardized for a decrement of 10 mg/dL in the low-density lipoprotein cholesterol level (to convert to millimoles per liter, multiply by 0.0259).

The findings between the *HMGCR* GRS and increased risk of Parkinson disease (OR, 1.30; 95% CI, 1.07-1.58; *P* = .007) and kidney failure (OR, 1.18; 95% CI, 1.05-1.34; *P* = .008) (Table 2) did not meet the Bonferroni-adjusted level of statistical significance (*P* < .002). In the prespecified sensitivity analysis, after adjusting for statin use (eTable 3 in the Supplement), the findings for Parkinson disease (OR, 1.30; 95% CI, 1.07-1.58; *P* = .007) and kidney failure (OR, 1.21; 95% CI, 1.07-1.37; *P* = .003) did not change markedly. Because type 2 diabetes is a common and important risk factor for developing kidney disease, we conducted a post hoc analysis and further adjusted the finding with kidney failure for the presence or absence of type 2 diabetes; was not statistically significant (OR, 1.10; 95% CI, 0.96-1.27; *P* = .16) (eTable 4 in the Supplement).

No other prespecified phenotypes were associated with the *HMGCR* GRS (Table 2). The *HMGCR* GRS was not associated with an increased risk of rhabdomyolysis, which is a well-documented statin adverse effect (OR, 1.38; 95% CI, 0.96-1.97; *P* = .08) (Table 2). The finding was not significant after adjustment for statin use (OR, 1.39; 95% CI, 0.97-1.99; *P* = .07) (eTable 3 in the Supplement).

We also compared individuals in different *HMGCR* GRS quartiles (Figure). Compared with those in the *HMGCR* GRS-estimated highest LDL-C level quartile, individuals in the lowest estimated LDL-C level quartile were more likely to have type 2 diabetes (OR, 1.11; 95% CI, 1.04-1.19; *P* = .001) (Figure, A) and the findings for kidney failure (OR, 1.26; 95% CI, 1.08-1.47; *P* = .004) (Figure, B) and Parkinson disease (OR, 1.44; 95% CI, 1.13-1.84; *P* = .003) (Figure, C) were not significant for the *P* value threshold of .002.

**Figure.**
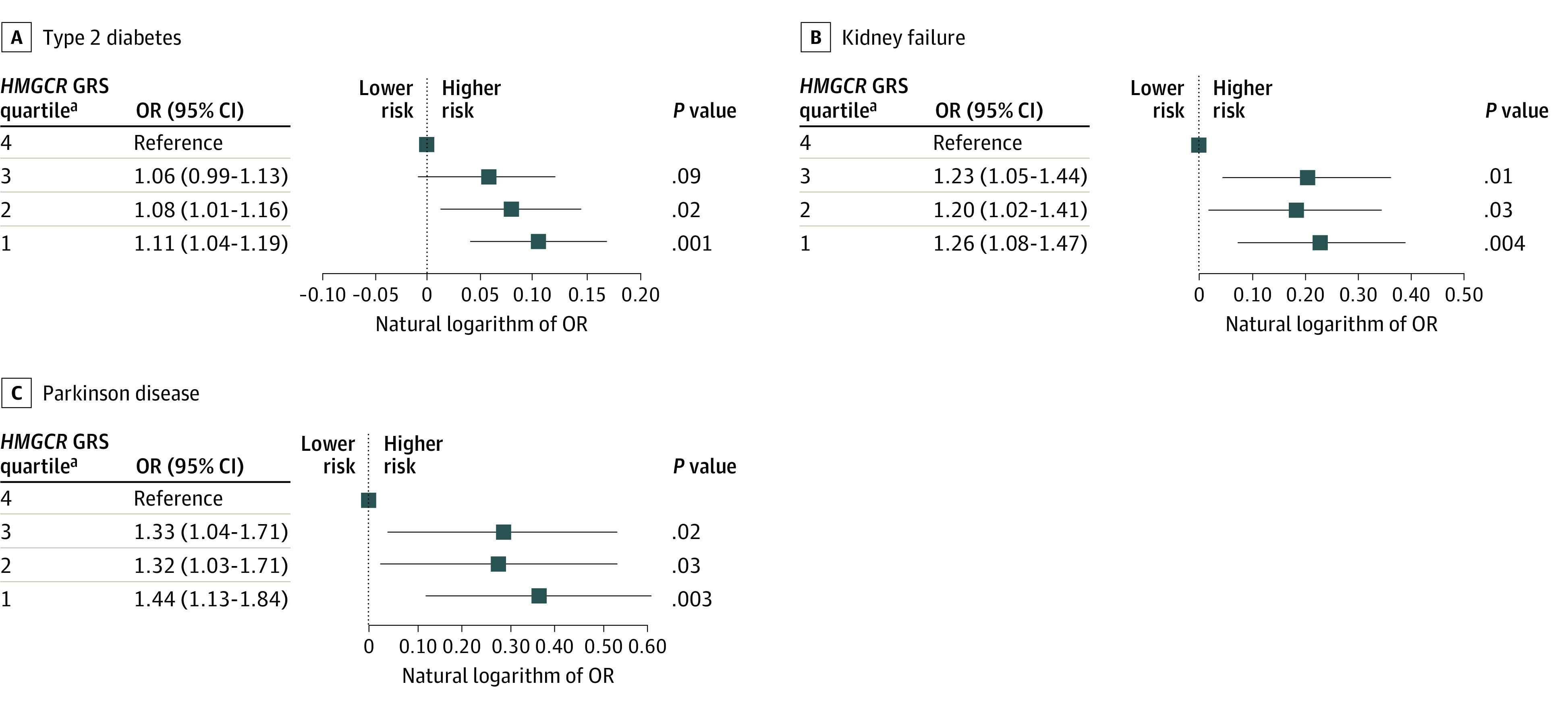
Association Between *HMGCR* Genetic Risk Score (GRS) Quartiles and Candidate Phenotypes ^a^Individuals in the lowest estimated LDL-C level quartile were more likely to have diseases.

### Replication in eMERGE Cohort

We calculated an *HMGCR* GRS for 30 444 unrelated adults of European ancestry in the eMERGE cohort (Table 1) and found that, for a 10-mg/dL *HMGCR* GRS–estimated decrease in the LDL-C level, the OR of having a diagnosis of hypercholesterolemia was 0.85 (95% CI, 0.77-0.92; *P* = 2.25 × 10^−4^) (eTable 2 in the Supplement). We tested *HMGCR* GRS and type 2 diabetes, kidney failure, and Parkinson disease in eMERGE. The finding with type 2 diabetes was statistically significant (OR, 1.09; 95% CI, 1.01-1.17; *P* = .02), and the findings for kidney failure (OR, 1.18; 95% CI, 0.98-1.41; *P* = .08) and Parkinson disease (OR, 0.93; 95% CI, 0.75-1.16; *P* = .53) (Table 3) were not significant. The ORs estimated for both type 2 diabetes and kidney failure in eMERGE (Table 3) were similar to those observed in BioVU (Table 2). In the prespecified sensitivity analysis, after adjusting for statin use in eMERGE (eTable 5 in the Supplement), the finding for type 2 diabetes was statistically significant (OR, 1.12; 95% CI, 1.04-1.21; *P* = .004), and the finding with kidney failure was significant (OR, 1.24; 95% CI, 1.03-1.49; *P* = .02). However, in the post hoc analysis, the finding between the *HMGCR* GRS and kidney failure was not statistically significant after adjusting for type 2 diabetes status (OR, 1.08; 95% CI, 0.88-1.32; *P* = .48) (eTable 4 in the Supplement).

**Table 3.** Findings Between *HMGCR* Genetic Risk Score and Candidate Phenotypes in Electronic Medical Records and Genomics Network^a^

PheCode	Description	*P* value	Odds ratio (95% CI)	No.	
				Cases	Controls
250.2	Type 2 diabetes	.02	1.09 (1.01-1.17)	6877	17 920
585.32	Kidney failure	.08	1.18 (0.98-1.41)	858	18 355
332	Parkinson disease	.53	0.93 (0.75-1.16)	538	19 330

^a^ The analyses were adjusted for sex, age at most recent visit, electronic health record length, and 5 principal components for ancestry. The *HMGCR* GRS was standardized for a decrement of 10 mg/dL in the low-density lipoprotein cholesterol level (to convert to millimoles per liter, multiply by 0.0259).

## Discussion

We used an *HMGCR* GRS as an instrumental variable for statin-induced lowering of the LDL-C level due to inhibition of HMG-CoA to evaluate statin treatment and 22 noncardiovascular diseases for which statins have been reported to have beneficial or detrimental outcomes in previous clinical trials or observational studies.^3^ The major finding of our study was that *HMGCR* GRS was not associated with the risk for most diseases; the reported association between statin treatment and the increased risk of type 2 diabetes was confirmed,^4,16^ and the next 2 strongest findings in BioVU—Parkinson disease and kidney failure—were not significant in eMERGE or were explained by type 2 diabetes status.

Although statins have been prescribed since 1987 and studied extensively, a modest increase in the risk of new-onset type 2 diabetes was only recognized in 2008.^17,18^ and has since been replicated in large-scale meta-analyses of randomized clinical trials.^18,19,20,21^ Reports from genetic analyses^4,16^ and well-controlled observational studies^22^ suggest that the increased type 2 diabetes risk is an on-target effect of statins: a result of a statin-induced decrease in the LDL-C level. By applying an MR approach in the EHR, we replicated the association between *HMGCR* and risk of type 2 diabetes and found that every 10-mg/dL estimated decrease of the LDL-C level was associated with an approximately 9% increase in the risk of type 2 diabetes—an estimate comparable with a previous MR report.^16^ However, the cardiovascular benefits of statins in clinical practice outweigh the small increased risk of type 2 diabetes. Nevertheless, particularly for patients with high type 2 diabetes risk (eg, patients with overweight or obesity), health care professionals could encourage a healthy diet and lifestyle and consider clinical monitoring for the development of type 2 diabetes.

The mechanisms underlying the increased risk of type 2 diabetes with statins are unclear; however, several hypotheses have been suggested. For example, in pancreatic cells, inhibition of cholesterol biosynthesis leads to impaired insulin secretion, and this mechanism can be reversed by cholesterol repletion^23^; also, statins can decrease insulin signaling through several mechanisms^24^ and decrease glucose uptake through reduced expression of glucose transporter 4.^24^

Because cholesterol is an essential constituent of the myelin encircling neurons in the brain, long-term low levels of LDL-C were thought to influence neuronal health and increase the risk of neurologic diseases, such as dementia and Parkinson disease.^25^ However, randomized clinical trials and observational studies have yielded inconsistent findings,^26,27,28,29^ and statins have been associated with both increased risk of cognitive dysfunction and decreased risk of dementia. Such disparate findings are likely due to residual confounding or indication bias. In the present study, we applied an MR approach to limit confounding and evaluated Parkinson disease and dementia. In BioVU, the finding between *HMGCR* GRS indicated probable lowering of the LDL-C levels and increased risk of Parkinson disease was not statistically significant. However, the Parkinson disease signal was also not significant in the eMERGE cohort. In a previous report, Benn et al^30^ used an MR approach with only 1 single-nucleotide variant from *HMGCR* and found no support for the proposed association between statin treatment and Parkinson disease.

Statins have generally been found to be renoprotective or neutral in studies of both acute kidney injury or progression of chronic kidney disease; a meta-analysis of 57 studies with 143 888 participants found no benefit of statin treatment on risk of kidney failure, but statins had small beneficial effects on the rate of decline in glomerular filtration rate and proteinuria.^31^ In contrast, a retrospective observational study using a propensity score–matched cohort of 6342 individuals receiving or not receiving statin therapy found an increased risk of both acute and chronic kidney disease in those receiving statin therapy.^32^ In the present study, the association between *HMGCR* GRS and kidney failure was close to statistical significance; however, this association was likely affected by confounding because it was not significant after adjusting for type 2 diabetes—a potent risk factor for kidney disease.

We found no significant association between the HMGCR GRS and increase risk of rhabdomyolysis. There was also no signal for myopathy, which is another muscle adverse effect commonly ascribed to statin. It is difficult to study muscle problems in EHRs because mild symptoms may not be well documented by diagnosis codes. Rhabdomyolysis is a well-documented but rare statin adverse effect; however, most cases of rhabdomyolysis, even in patients who receive statin therapy, are due to surgery, trauma, or other factors rather than statins.^33^ It remains controversial whether rhabdomyolysis is an off-target or on-target statin adverse effect. Both genetic and observational studies of rhabdomyolysis are difficult to conduct because the condition is rare. A study in a very large cohort would be needed to elucidate the association between statin use and rhabdomyolysis.

### Strengths and Limitations

This study has several strengths. First, we conducted a comprehensive evaluation of 22 candidate phenotypes in 2 large cohorts: BioVU and eMERGE. Second, we applied an MR approach using genetic variants in the *HMGCR* gene, which is a method that is less susceptible to confounding and reverse causation than observational studies. Although the *HMGCR* GRS explains only a relatively small amount of variation in LDL-C levels, it is a genetic instrument whose utility has been demonstrated.^16^

This study also has several limitations. First, in addition to associations with diseases, statins have been reported to affect mortality related to diseases such as cancer. Disease-specific mortality is difficult to study in the EHR; thus, we did not include such outcomes. Second, we constructed an *HMGCR* GRS based on the associations of genetic variants with LDL-C levels as an instrumental variable for statins; however, we may not detect off-target statin effects (ie, statin effects that are not mediated by decreasing LDL-C levels). Until the genetic determinants of statin effects unrelated to their decreases in LDL-C levels are characterized it will not be possible to use an MR approach to address the potential off-target risks or benefits of statin therapy. Third, we did not test the differential outcomes of statins in patients who received them at different ages because MR uses a GRS that represents lifelong risk. Fourth, for 12 phenotypes in BioVU, we had more than 1000 cases, but for uncommon phenotypes, such as rhabdomyolysis (n = 159), our ability to detect a genetic signal was limited. Fifth, we focused on individuals of European ancestry and do not know whether the observations apply to other racial/ethnic groups because the prevalence of noncardiovascular outcomes (eg, type 2 diabetes) varies between different ancestries and the association between *HMGCR* variant alleles and LDL-C may differ in populations of varying ancestry.^34^

## Conclusions

We applied an MR approach in this cohort study using genetic variants in the *HMGCR* gene that affect LDL-C levels and assessed GRS and candidate phenotypes in 2 large cohorts to evaluate disease and modulation of HMG-CoA activity. We replicated the finding between statin use and increased risk of type 2 diabetes, but there was no strong indication of other pleiotropic outcomes of statins related to their LDL-C level–decreasing effects.

## Notes

Supplement. **eMethods.** Genotype Quality Control and Imputation eReferences **eTable 1.** List of Genetic Variants in the *HMGCR* GRS **eTable 2.** Associations Between *HMGCR* GRS and Hypercholesterolemia **eTable 3.** Sensitivity Analysis of Associations Between *HMGCR* GRS and Candidate Phenotypes in BioVU Adjusted for Statin Use **eTable 4.** Sensitivity Analysis for Association Between *HMGCR* GRS and End Stage Renal Disease, Further Adjusting Type 2 Diabetes Status **eTable 5.** Sensitivity Analysis of Associations Between *HMGCR* GRS and Candidate Phenotypes in eMERGE Adjusting for Statin Use **eFigure.** Associations Between *HMGCR* GRS Quartiles and Hypercholesterolemia
