# Supplementary material for: A Mendelian Randomization Approach Using 3-HMG-Coenzyme-A Reductase Gene Variation to Evaluate the Association of Statin-Induced Low-Density Lipoprotein Cholesterol Lowering With Noncardiovascular Disease Phenotypes
Source: JAMA Netw Open. 2021 Jun 7;4(6):e2112820. doi: 10.1001/jamanetworkopen.2021.12820 (PMC8185593; doi:10.1001/jamanetworkopen.2021.12820)
Supplement: Supplement. — eMethods. Genotype Quality Control and Imputation eReferences eTable 1. List of Genetic Variants in the HMGCR GRS eTable 2. Associations Between HMGCR GRS and Hypercholesterolemia eTable 3. Sensitivity Analysis of Associations Between HMGCR GRS and Candidate Phenotypes in BioVU Adjusted for Statin Use eTable 4. Sensitivity Analysis for Association Between HMGCR GRS and End Stage Renal Disease, Further Adjusting Type 2 Diabetes Status eTable 5. Sensitivity Analysis of Associations Between HMGCR GRS and Candidate Phenotypes in eMERGE Adjusting for Statin Use eFigure. Associations Between HMGCR GRS Quartiles and Hypercholesterolemia [file jamanetwopen-e2112820-s001.pdf]

Supplementary Online Content

Liu G, Shi M, Mosley JD, et al. A mendelian randomization approach using 3-HMG-coenzyme-A-reductase gene variation to evaluate the association of statin-induced low-density lipoprotein cholesterol lowering with noncardiovascular disease phenotypes. *JAMA Netw Open*. 2021;4(6):e2112820. doi:10.1001/jamanetworkopen.2021.12820

eMethods. Genotype Quality Control and Imputation

eReferences

eTable 1. List of Genetic Variants in the *HMGCR* GRS

eTable 2. Associations Between *HMGCR* GRS and Hypercholesterolemia

eTable 3. Sensitivity Analysis of Associations Between *HMGCR* GRS and Candidate Phenotypes in BioVU Adjusted for Statin Use

eTable 4. Sensitivity Analysis for Association Between *HMGCR* GRS and End Stage Renal Disease, Further Adjusting Type 2 Diabetes Status

eTable 5. Sensitivity Analysis of Associations Between *HMGCR* GRS and Candidate Phenotypes in eMERGE Adjusting for Statin Use

eFigure. Associations Between *HMGCR* GRS Quartiles and Hypercholesterolemia

This supplementary material has been provided by the authors to give readers additional information about their work.

© 2021 Liu G et al. *JAMA Network Open.*

eMethods

Genotype quality control and imputation

BioVU samples were genotyped on the Infinium Multi-Ethnic Genotyping Array (MEGA). Genotyping data was curated for quality control using PLINK 1.9.[1] We removed the following samples: (1) with per-individual call rate <95%; (2) with wrongly assigned sex; (3) related individuals (PI_HAT ≥ 0.25);[2] (4) unexpected duplications. To increase coverage of the GWAS, we performed genome imputation using the Michigan Imputation Server)[3,4] with the Haplotype Reference Consortium [5] as reference. Principal components (PCs) for ancestry were calculated using common variants (MAF>1%) with high variant call rate (>98%), excluding variants in linkage and regions known to affect PCs (HLA region on chromosome 6, inversion on chromosome 8 (8135000-12000000) and inversion on chromosome 17 (40900000-45000000, GRCh37 build). We calculated 10 PCs for ancestry using SNPRelate v1.16.0.[6]

Genotype data in eMERGE were acquired on the Illumina Human660W-Quadv1_A, HumanOmni1-Quad, HumanOmni5-Quad, MEGA-EX, Human610, Human550, HumanOmniExpressExome-8v1.2A, and MegArray platforms. Quality control steps for eMERGE were performed following previously published protocols[7] using PLINK version 1.9.[8] SNPs were pre-phased using SHAPEIT [9] and imputed using IMPUTE2 version 2.3.0, [10] with 1000 Genomes as reference. Imputed data were filtered for call rate >98% and SNP deviation from Hardy-Weinberg $P < 10^{-6}$. We calculated PCs using the same approach as in BioVU (SNPRelate package).[6]

© 2021 Liu G et al. *JAMA Network Open.*

eReferences

1. Purcell S, Neale B, Todd-Brown K, et al. PLINK: a tool set for whole-genome association and population-based linkage analyses. *Am J Hum Genet*. 2007;81(3):559-575. doi:S0002-9297(07)61352-4 [pii] 10.1086/519795

2. Hancock DB, Eijgelsheim M, Wilk JB, et al. Meta-analyses of genome-wide association studies identify multiple loci associated with pulmonary function. *Nat Genet*. 2010;42(1):45-52. doi:10.1038/ng.500

3. Das S, Forer L, Schönherr S, et al. Next-generation genotype imputation service and methods. *Nature Genetics*. 2016;48(10):1284. doi:10.1038/ng.3656

4. Michigan Imputation Server. Accessed March 11, 2021. https://imputationserver.sph.umich.edu/index.html#!

5. McCarthy S, Das S, Kretzschmar W, et al. A reference panel of 64,976 haplotypes for genotype imputation. *Nat Genet*. 2016;48(10):1279-1283. doi:10.1038/ng.3643

6. X Z, D L, J S, Sm G, C L, Bs W. A High-Performance Computing Toolset for Relatedness and Principal Component Analysis of SNP Data. Bioinformatics (Oxford, England). doi:10.1093/bioinformatics/bts606

7. Rl Z, Ll A, Sj B, et al. Pitfalls of Merging GWAS Data: Lessons Learned in the eMERGE Network and Quality Control Procedures to Maintain High Data Quality. Genetic epidemiology. doi:10.1002/gepi.20639

8. Chang CC, Chow CC, Tellier LC, Vattikuti S, Purcell SM, Lee JJ. Second-generation PLINK: rising to the challenge of larger and richer datasets. *Gigascience*. 2015;4:7. doi:10.1186/s13742-015-0047-8

9. Delaneau O, Zagury J-F, Marchini J. Improved whole-chromosome phasing for disease and population genetic studies. *Nat Methods*. 2013;10(1):5-6. doi:10.1038/nmeth.2307

10. Howie B, Fuchsberger C, Stephens M, Marchini J, Abecasis GR. Fast and accurate genotype imputation in genome-wide association studies through pre-phasing. *Nat Genet*. 2012;44(8):955-959. doi:10.1038/ng.2354

11. Ference BA, Robinson JG, Brook RD, et al. Variation in PCSK9 and HMGCR and Risk of Cardiovascular Disease and Diabetes. *New England Journal of Medicine*. 2016;375(22):2144-2153. doi:10.1056/NEJMoa1604304

© 2021 Liu G et al. *JAMA Network Open.*

eTable 1. List of genetic variants in the HMGCR GRS (adapted from Ference et al.)[11]

SNP	Position(hg19)	Position(hg18)	effect_allele	other_allele	beta	SE
rs12916	5:74656539	5:74692295	T	C	2.3456	0.1216
rs17238484	5:74648496	5:74684252	G	T	2.0064	0.1984
rs5909	5:74656175	5:74691931	G	A	1.9744	0.2816
rs2303152	5:74641707	5:74677463	G	A	1.3536	0.2048
rs10066707	5:74560579	5:74596335	G	A	1.5904	0.1728
rs2006760	5:74562029	5:74597785	C	G	1.7056	0.2432

© 2021 Liu G et al. *JAMA Network Open.*

eTable 2. Associations between HMGCR GRS and hypercholesterolemia							
(A) BioVU							
				95% CI			
**Phecode**	**Description**	**P-value**	**Odds Ratio**	**LCI**	**UCI**	**n_cases**	**n_controls**
272.11	Hypercholesterolemia	0.009	0.91	0.85	0.98	6523	29914
(B) eMERGE							
				95% CI			
**PheCode**	**Description**	**P-value**	**Odds Ratio**	**LCI**	**UCI**	**n_cases**	**n_controls**
272.11	Hypercholesterolemia	2.25E-04	0.85	0.77	0.92	9969	8811

* The analyses have been adjusted for gender, age at most recent visit, EHR length, and 10 PCs(BioVU) or 5 PCs (eMERGE) for ancestry.
We standardized the HMGCR GRS for a decrement of 10 mg/dl LDL-C

© 2021 Liu G et al. *JAMA Network Open.*

eTable 3. Sensitivity analysis of associations between HMGCR GRS and candidate phenotypes in BioVU adjusted for statin use									
				95% CI					
PheCode	Description	P-value	OR	LCI	UCI	n_total	n_cases	n_controls
250.2	Type 2 diabetes	8.50E-06	1.13	1.07	1.19	47757	9210	38547
585.32	End stage renal disease	0.003	1.21	1.07	1.37	39811	1308	38503
332	Parkinson's disease	0.007	1.30	1.07	1.58	41193	553	40640
772.4	Rhabdomyolysis	0.07	1.39	0.97	1.99	46140	159	45981
202.2	Non-Hodgkins lymphoma	0.11	1.21	0.96	1.52	51062	367	50695
151	Cancer of stomach	0.12	0.86	0.71	1.04	45818	509	45309
770	Myalgia and myositis unspecified	0.20	1.05	0.97	1.14	50460	3202	47258
800.1	Fracture of neck of femur	0.23	1.11	0.94	1.32	45613	679	44934
585.1	Acute renal failure	0.27	1.03	0.97	1.10	44949	6446	38503
38	Septicemia	0.29	1.05	0.96	1.13	46724	3049	43675
185	Cancer of prostate	0.30	0.94	0.84	1.05	18262	1786	16476
577.1	Acute pancreatitis	0.31	1.10	0.92	1.31	51731	596	51135
634	Miscarriage; stillbirth	0.38	0.88	0.65	1.18	27815	234	27581
366.2	Senile cataract	0.47	1.03	0.96	1.10	50734	4469	46265
8.52	Intestinal infection due to C. difficile	0.48	0.94	0.80	1.11	51807	672	51135
70.3	Viral hepatitis C	0.53	0.96	0.84	1.09	44776	1168	43608
290.1	Dementias	0.61	1.04	0.89	1.21	42515	890	41625
153.2	Colon cancer	0.72	0.98	0.87	1.10	43091	1510	41581
155.1	Malignant neoplasm of liver, primary	0.91	1.01	0.83	1.24	45770	461	45309
743.11	Osteoporosis	0.95	1.00	0.92	1.09	45863	3410	42453
38.3	Bacteremia	0.95	1.00	0.90	1.10	45543	1868	43675

© 2021 Liu G et al. *JAMA Network Open.*

359.2	Myopathy	0.98	1.00	0.87	1.14	47572	1028	46544

* The analyses have been adjusted for gender, age at most recent visit, EHR length, 10 PCs for ancestry, and statin use; Cancer of prostate was analyzed only in male; Miscarriage or stillbirth was analyzed only in female.
We standardized the *HMGCR* GRS for a decrement of 10 mg/dl LDL-C

© 2021 Liu G et al. *JAMA Network Open.*

eTable 4. sensitivity analysis for association between HMGCR wGRS and end stage renal disease, further adjusting type 2 diabetes status

(A) BioVU

				**95% CI**			
**PheCode**	**Description**	**P-value**	**Odds Ratio**	**LCI**	**UCI**	**n_cases**	**n_controls**
585.32	End stage renal disease	0.16	1.10	0.96	1.27	1102	34976

(B) eMERGE

				**95% CI**			
**PheCode**	**Description**	**P-value**	**Odds Ratio**	**LCI**	**UCI**	**n_cases**	**n_controls**
585.32	End stage renal disease	0.48	1.08	0.88	1.32	725	15923

* The analyses have been adjusted for gender, age at most recent visit, EHR length, 10 PCs(BioVU) or 5 PCs (eMERGE) for ancestry, statin use, and type 2 diabetes status.
We standardized the HMGCR GRS for a decrement of 10 mg/dl LDL-C

© 2021 Liu G et al. *JAMA Network Open.*

eTable 5. Sensitivity analysis of associations between HMGCR GRS and candidate phenotypes in eMERGE adjusting for statin use

				95% CI			
**PheCode**	**Description**	**P-value**	**Odds Ratio**	**LCI**	**UCI**	**n_cases**	**n_controls**
250.2	Type 2 diabetes	0.004	1.12	1.04	1.21	6877	17920
585.32	End stage renal disease	0.02	1.24	1.03	1.49	858	18355
332	Parkinson's disease	0.52	0.93	0.75	1.16	538	19330

* The analyses have been adjusted for gender, age at most recent visit, EHR length, statin use and 5 PCs for ancestry. We standardized the *HMGCR* GRS for a decrement of 10 mg/dl LDL-C

© 2021 Liu G et al. *JAMA Network Open.*

eFigure. Associations between HMGCR GRS quartiles and hypercholesterolemia.

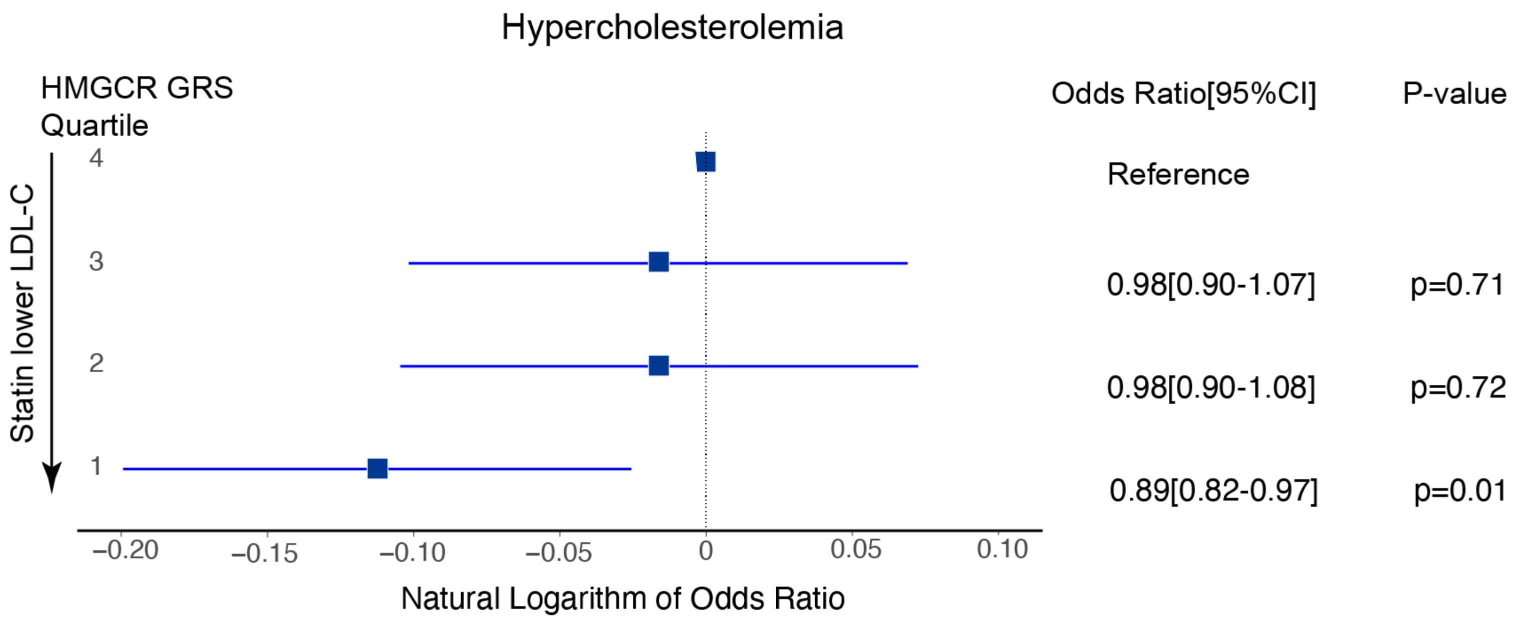

© 2021 Liu G et al. *JAMA Network Open.*
